# Eye movement characteristics provide an objective measure of visual processing changes in patients with visual snow syndrome

**DOI:** 10.1038/s41598-021-88788-2

**Published:** 2021-05-05

**Authors:** Emma J. Solly, Meaghan Clough, Allison M. McKendrick, Paige Foletta, Owen B. White, Joanne Fielding

**Affiliations:** 1 Department of Neuroscience, Central Clinical School, Monash University, 6th Floor, The Alfred Centre, 99 Commercial Road, Melbourne, VIC 3004 Australia; 2 Department of Optometry and Vision Sciences, The University of Melbourne, Melbourne, VIC Australia; 3 Department of Medicine, Royal Melbourne Hospital, The University of Melbourne, Parkville, VIC Australia

**Keywords:** Neuroscience, Cognitive neuroscience, Oculomotor system, Sensory processing, Visual system

## Abstract

Visual snow syndrome (VSS) is a poorly understood neurological disorder that features a range of disabling sensory changes. Visual processing changes revealed previously in VSS appear consistent with poor attentional control, specifically, with difficulty controlling environmentally driven shifts of attention. This study sought to confirm this proposal by determining whether these changes were similarly evident where attention is internally driven. Sixty seven VSS patients and 37 controls completed two saccade tasks: the endogenously cued saccade task and saccadic Simon task. The endogenously cued saccade task correctly (valid trial) or incorrectly (invalid trial) pre-cues a target location using a centrally presented arrow. VSS patients generated significantly shorter saccade latencies for valid trials (*p* = 0.03), resulting in a greater magnitude *cue effect* (*p* = 0.02), i.e. the difference in latency between valid and invalid trials. The saccadic Simon task presents a peripheral cue which may be spatially congruent or incongruent with the subsequent target location. Latencies on this task were comparable for VSS patients and controls, with a normal *Simon effect*, i.e. shorter latencies for saccades to targets spatially congruent with the preceding cue. On both tasks, VSS patients generated more erroneous saccades than controls towards non-target locations (Endogenously cued saccade task: *p* = 0.02, saccadic Simon task: *p* = 0.04). These results demonstrate that cued shifts of attention differentially affect saccade generation in VSS patients. We propose that these changes are not due to impairment of frontally-mediated inhibitory control, but to heightened saccade-related activity in visual regions. These results contribute to a VSS ocular motor signature that may provide clinical utility as well as an objective measure of dysfunction to facilitate future research.

## Introduction

Visual snow syndrome (VSS) is a poorly understood neurological disorder characterized by a range of debilitating visual symptoms, including visual snow (VS), a persistent perceptual phenomenon that manifests as a veil of flickering dots across the entire visual field^1^. Non-visual sensory symptoms commonly co-occur with VSS, including tinnitus, paraesthesia, and migraine^2,3^. Initially assumed to be a persistent variant of migraine visual aura, VSS is now recognised as a distinct but overlapping disorder. In contrast to migraine aura, which is generally a short-lived visual disturbance preceding a migraine, VS is continuous, and phenotypically distinct from typical descriptions of migraine aura^3,4^. Further, a significant number of VSS patients do not experience migraine^5^. Onset of VSS is typically in the second or third decade of life, often without a clear triggering event^6^, although approximately 40% of patients report having always experienced VSS^5,6^. Standard ophthalmic and neurological examinations, including MRI, are typically normal^3^.

At present, the pathophysiology of VSS remains unknown and there are no objective measures of the disorder, hampering the provision of effective treatments^7^. However, the diverse range of associated visual and other sensory symptoms suggest a central disturbance in the processing of sensory information. Proposed mechanisms include cortical hyperexcitability^4,8^, and/or dysfunction of thalamocortical networks associated with sensory processing^9,10^. These theories are supported by a few small scale behavioural^11,12^, electrophysiological^9,10^, and imaging studies^6,13^.

Given the significant functional and physiological overlap between visual processing and saccade networks in the brain, ocular motor tasks may be used to evaluate the integrity of networks underpinning visual processing impairments^12,14,15^. Indeed, our own preliminary work revealed ocular motor changes in VSS that included significantly faster eye movements towards suddenly appearing visual stimuli and difficulty inhibiting erroneous eye movements, i.e. increased antisaccade errors^12^. As no evidence of executive impairment was found when participants were required to switch randomly between a simple and more difficult saccadic task, we determined that the increased error rate was likely attributable to the faster visually-guided response^16^. However, we propose that these changes might also be viewed as a more rapid stimulus-driven (exogenous) shift of attention, with the altered excitability of visual cortical regions in VSS resulting in heightened reactivity to visual stimuli and a correspondingly faster saccade.

Importantly, attention is not only captured exogenously, but also directed endogenously, in line with internal goals and motivations. Here we investigated whether ocular motor changes are evident in VSS patients when attention is endogenously (internally) directed rather than exogenously captured. Two classic ocular motor tasks were implemented: the endogenously cued saccade task and the saccadic Simon task. The endogenously cued saccade task is based on the classic Posner paradigm^17^, and examines the effect of manipulating attentional set by presenting a directional cue (i.e. arrow) prior to the appearance of a visual target. The saccadic Simon task examines the effect of presenting an informative cue (i.e. shape) that is either spatially congruent or incongruent with the target location^18^; participants are required to endogenously shift attention towards, and interpret the cue, prior to making an eye movement.

This study aimed to determine whether increased cortical excitability in VSS impacts endogenous shifts of attention similarly to exogenous shifts of attention, resulting in an increased rate of erroneous saccades to non-target stimuli for both saccade tasks, and shorter latencies for visually guided saccades for the endogenously cued saccade task. We propose that the results herein will contribute to a VSS ocular motor signature that may be used to both further research the neuropathological underpinnings of the disorder, and assist with patient management, in particular, assessment of the efficacy of future treatments. This goal is particularly important given the current lack of objective measures of VSS.

## Materials and methods

### Participants

We recruited 67 patients who met the criteria for VSS as specified by the International Classification of Headache Disorders (ICHD-3: see Table 1) through a combination of online, radio, and television advertising.

**Table 1:** International classification of headache disorders (ICHD-3) criteria for a diagnosis of visual snow

A. Visual snow: dynamic, continuous tiny dots across the entire visual field, persisting for > 3 months	
B. Additional visual symptoms of at least two of the following four types:	
1. Palinopsia	
2. Enhanced entoptic phenomena	
3. Photophobia	
4. Impaired night vision (nyctalopia)	
C. Symptoms are not consistent with typical migraine visual aura	
D. Symptoms are not better accounted for by another disorder	

Ophthalmic examination revealed normal visual acuity, colour vision, and retinal anatomy. Thirty seven healthy control participants were recruited from the community. Exclusion criteria for all participants were having experienced a migraine or migraine aura within 3 days preceding or following testing, a confounding neurological or visual condition, or consumption of any medication likely to affect cognitive or visual function.

Participant (control and VSS) numbers differed across tasks due to personal and technical issues. Although VSS patients reported higher depression, anxiety, and stress scores overall on the Depression Anxiety Stress Scale (DASS), *F*(1, 80)=− 5.91, *p*<0.001), VSS and control groups did not differ in age, measures of substance use/abuse (Drug Use Disorder Identification Test- DUDIT^19^, Alcohol Use Disorders Identification Test- AUDIT^20^), or estimated premorbid intelligence (National Adult Reading Test: NART^21^). DASS, DUDIT, AUDIT, and NART scores did not correlate with any saccadic variables. Demographic information for all participants is displayed in Table 2.

**Table 2:** Demographic information for all participants.

	VSS *Mean* (SD) *n* = *32*	VSS + Migraine *Mean* (SD) *n* = 35	Controls *Mean* (SD) *n* = 37
Female/Male	12/20	24/11	24/12
Age/distribution	29.38/18–54	31.78/20–55	27.56/18–56
Visual snow			
Duration (years)	14.81 (13.3)	13.42 (11.72)	
Patients with lifelong duration (%)	39.3	34.4	
Afterimages (%)	67.9	93.1	
Photophobia (%)	46.4	62.1	
Nyctalopia (%)	75	58.6	
Floaters (%)	82.1	86.2	
Blue field entoptic phenomenon (%)	67.9	79.3	
Tinnitus (%)	67.9	67.9	
Paraesthesia (%)	25	42.9	
Family history of migraine (%)	30.4	59.3	
Relative with VS (%)	4.3	7.4	
DASS			
Depression	12.42 (9.84)	8.41 (8.62)	2.62 (3.72)
Anxiety	6.33 (4.87)	7.07 (7.5)	1.86 (2.2)
Total	10.53 (6.69)	8.57 (6.8)	2.92 (3.24)
AUDIT	3.17 (4.36)	2.45 (1.59)	2.62 (1.57)
DUDIT	1.21 (2.93)	1.24 (3.63)	.52 (1.72)
NART	113.88 (5.95)	114.17 (4.87)	116.15 (5.65)

*VSS* visual snow syndrome, *DASS* depression anxiety stress scale, *AUDIT* alcohol use disorders identification test, *DUDIT* drug use disorder identification test, *NART* national adult reading test.

### Standard protocol approvals, registrations, and patient consents

Ethics approval was granted by the Monash University Human Research Ethics Committee, and all participants provided written informed consent prior to participation in the study. All methods were performed in accordance with the declaration of Helsinki.

### Equipment, stimuli, and procedures

The following ocular motor tasks were conducted at the Alfred Centre, Monash University, Melbourne, over a one hour session. For each task, the latency and accuracy of saccades was measured using an Eyelink 1000 plus dark pupil video-oculography system. This system has a high resolution (noise limited at <0.01°), an acquisition rate of 1000 Hz, and a refresh rate of 60Hz. Participants were seated in a darkened room, on a height-adjustable chair, with a forehead and chin rest used to stabilise the head during recordings. This situated the participant’s eye 950mm from a monitor (resolution:1920 × 1080). Task stimuli were displayed on a black background, and calibration was performed using a five-point grid as per the built-in Eyelink process.

#### Endogenously cued saccade task

**Figure 1:**
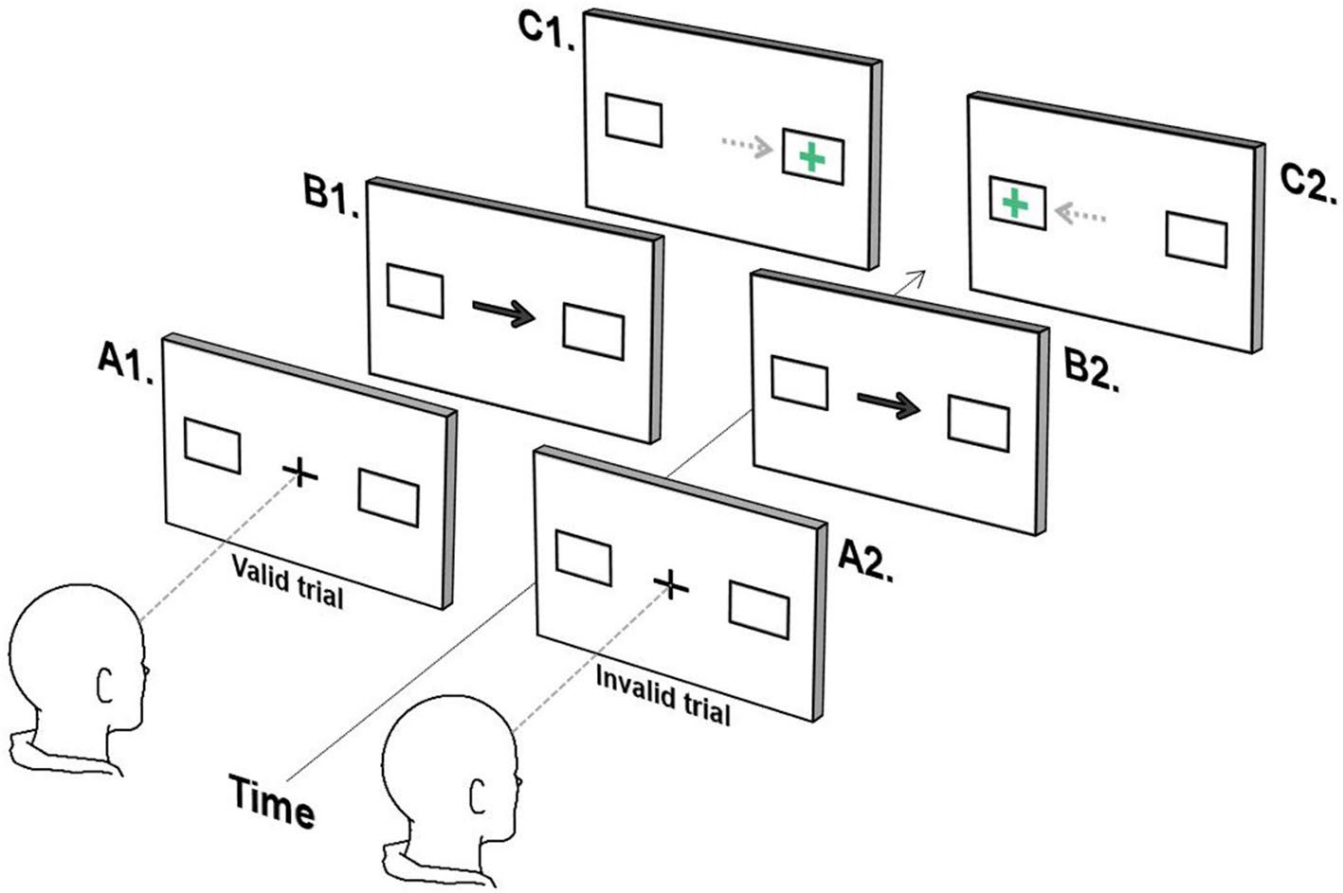
Endogenously cued saccade task. (**A**) Participants fixate on the central cross, which is replaced by an arrow indicating the left or right box (**B**). The participant must remain fixated on the centre until a cross appears either in the box indicated by the arrow (**C**1, valid trial), or in the opposite box (**C**2, invalid trial) at which point they look towards the cross.

This task derives from Posner’s attentional orienting task and explores the impact, on latency, of endogenously redirecting attention prior to target presentation^17^. Participants were instructed to fixate a central green cross (1.5°) flanked by 2 white boxes presented 10 degrees right and left of centre. Following 400 ms, the central cross was replaced by an arrow (1.5°) for 500 ms, pointing to either the left or right box. Participants were instructed to ‘ignore’ the central arrow and maintain fixation at centre until a green cross appeared in the box either indicated by the arrow (valid trial) or in the opposite direction (invalid trial). As soon as the green cross appeared, participants were required to look directly towards it. Participants were familiarised with the task by way of a guided example (see Figure 1).

The task comprised 120 trials (2 blocks of 60 trials), 75% valid trials and 25% invalid trials presented in a random order. The preponderance of valid trials typically creates the expectation that the central arrow correctly predicts the location of the upcoming green cross^17^. A ‘cue effect’ is subsequently observed, where latencies for valid trials are significantly shorter than latencies for invalid trials.

#### Saccadic Simon task

**Figure 2:**
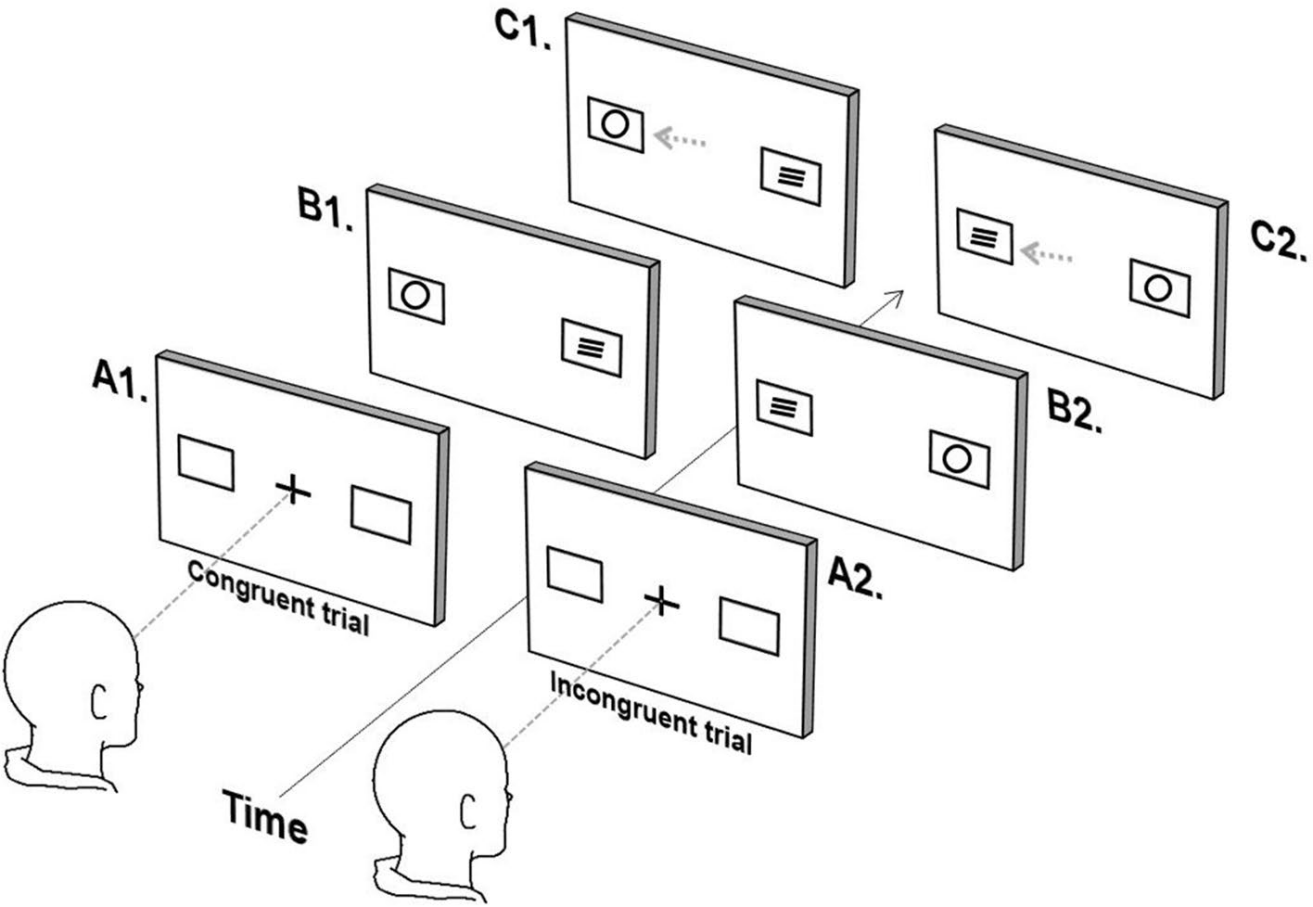
Saccadic Simon task (only circles trials depicted). (**A**) Participants fixates on the central cross, and (**B**) a shape (circle/square) appears concomitant with the appearance of a ‘filler symbol’ (three horizontal lines) in the opposite box. The participant determines the direction of the eye movement based on the shape (circle = leftward movement, square = rightwards movement) and (**C**) performs an eye movement to the corresponding box. (**B**1/**C**1) depict a congruent trial, and (**B**2/**C**2) depict an incongruent trial.

Participants were instructed to fixate a central white cross flanked by 2 white boxes presented 10 degrees to the right/left of centre. Following 1000 ms, either a ‘circle’ or a ‘square’ was presented in one of the peripheral boxes and a filler stimulus (three stacked horizontal lines) presented in the opposite box. The appearance of a circle in either box indicated that the participant should look immediately towards the box on the left hand side, while the appearance of a square in either box indicated that the participant should look immediately towards the box on the right hand side. Shape and filler stimuli were presented for 3000 ms. Trials were either congruent, if the target location and trial stimulus (circle/square) were in the same location, or incongruent, if the target location and trial stimulus were in opposite locations (see Figure 2).

Typically, the latency of an incongruent trial is longer than a congruent trial; this is known as the ‘Simon effect’, although is seen only on those trials following a correctly executed congruent trial^22^. For analyses, trials were classified according to the congruency of both the previous and current trial. The task comprised 128 trials (4 blocks of 32 trials), with an equal number of each trial type presented in a pseudo-random order. Participants were familiarised with the task with by way of a guided example and a practice block consisting of 10 trials (5 congruent, 5 incongruent).

### Data analysis

Analyses of eye movement data were performed offline using a custom Matlab program. Saccade latency (ms) was calculated as the temporal difference between stimulus onset (endogenously cued saccade task: green cross, saccadic Simon task: circle/square) and saccade onset. Saccade onset was indicated by a visually evident departure from baseline corresponding to a change in the velocity profile of the saccade trace of 30° per second. Trials were excluded from latency analysis where an error was performed (see below), fixation was not maintained within 2° of centre, a blink occurred following trial onset, or no response was made.

For the endogenously cued saccade task, latencies were calculated separately for valid and invalid trials. A cue effect was calculated (invalid minus valid trial latencies), and an error rate as the proportion of errors to the total number of trials; an error describes an incorrect saccade exceeding 1.5 degrees in the direction indicated by the central arrow, either prior to or within 100ms following target presentation.

For the saccadic Simon task, latencies were calculated separately for all trial types. Given that congruency effects are contingent upon performance in the previous trial^22^, trials that directly followed an error were not included in latency analysis. An error was defined as a saccade exceeding 1.5° in the incorrect direction following appearance of the trial stimulus, e.g. a rightward saccade following the appearance of a circle: error rate was calculated as the proportion of errors to the total number of trials. The Simon effect was calculated as incongruent trial latency minus congruent trial latency, for those trials following a correctly executed congruent trial.

### Statistical analyses

Data analyses were conducted using IBM SPSS Statistics 26^23^. An alpha-level of *p* < 0.05 was set for all analyses, and where data was missing, pairwise deletion was used. Repeated measures ANOVA were conducted for both tasks: endogenously cued saccade latency: group (controls, VSS) × trial type (valid, invalid); saccadic Simon task latency and error rate: group (controls, VSS) × previous trial type (congruent, incongruent) × current trial type (congruent, incongruent). Post hoc analyses used either t-tests or one-way ANOVAs. Since errors on the endogenously cued task occurred prior to target presentation (thus prior to the determination of trial type), error rate was compared between groups using a one-way ANOVA. A ROC curve analysis was performed for variables where VSS patients differed significantly from controls: valid latencies on the endogenously cued saccade task, and total error rates for both tasks. Benjamini–Hochberg corrections were made for all multiple comparisons^24^.

## Results

Although we acknowledge that there may be subtle visual symptoms associated with migraine itself (independent of headache), we revealed no significant differences between VSS patients with migraine (n = 35) and those without migraine (n = 32) on any experimental variable (see Table 3). As such all patients were combined into a single VSS group. This is consistent with our previous study^12^. Mean latencies and error rates for controls and the combined VSS patient group can be found in Table 4.

**Table 3:** VSS group means and standard deviations for ocular motor task variables.

	VSS no migraine *Mean* (SD) *n* = 32	VSS + Migraine *Mean* (SD) *n* = 35	*p*
Endogenously cued saccade latencies (ms)			
Valid trials	234.48 (33.21)	235.33 (24.56)	0.909
Invalid trials	247.09 (49.42)	248.58 (28.01)	0.884
Endogenously cued saccade errors (%)	6.61 (7.68)	6.9 (6.63)	0.874
Saccadic Simon task latency (ms)	492.63 (13.12)	517.68 (57.25)	0.128
Saccadic Simon task total errors (%)	15.97 (9.21)	12.43 (6.71)	0.064

*VSS* visual snow syndrome.

**Table 4:** Means and standard deviations for ocular motor task variables.

	Control *Mean* (SD)	VSS *Mean* (SD)	*p*
Endogenously cued saccade latencies (ms)	*n* = 36	*n* = 60	
Valid trials	248.86 (31.65)	234.94 (28.67)	**0.029**
Invalid trials	253.16 (36.18)	247.89 (39.12)	0.513
Cue effect	4.3 (12.7)	12.95 (23.16)	**0.02**
Endogenously cued saccade errors (%)	3.66 (4.57)	6.76 (7.08)	**0.01**
Saccadic Simon task latencies (ms)	*n* = 28	*n* = 66	
Congruent-congruent trials	490.64 (89.76)	495.1 (69.84)	0.796
Congruent-incongruent trials	502.16 (85.82)	518.88 (73.88)	0.342
Incongruent-congruent trials	497.71 (89.29)	499.98 (72.56)	0.897
Incongruent-incongruent trials	492.24 (77.64)	508.18 (72.8)	0.343
Simon effect (ms)	11.51 (32.37)	23.78 (46.46)	0.207
Saccadic Simon task errors (%)			
Congruent-congruent trials	7.9 (6.14)	13.22 (8.73)	**0.004**
Congruent-incongruent trials	15.27 (11.7)	18.65 (12.04)	0.212
Incongruent-congruent trials	10.97 (6.94)	14.88 (9.51)	0.053
Incongruent-incongruent trials	9.34 (11.01)	12.25 (10.8)	0.239

*VSS* visual snow syndrome.

Underline: indicates the trial of interest. **Bold**: significant at the .05 probability level.

### Endogenously cued saccade task

#### Latency

**Figure 3:**
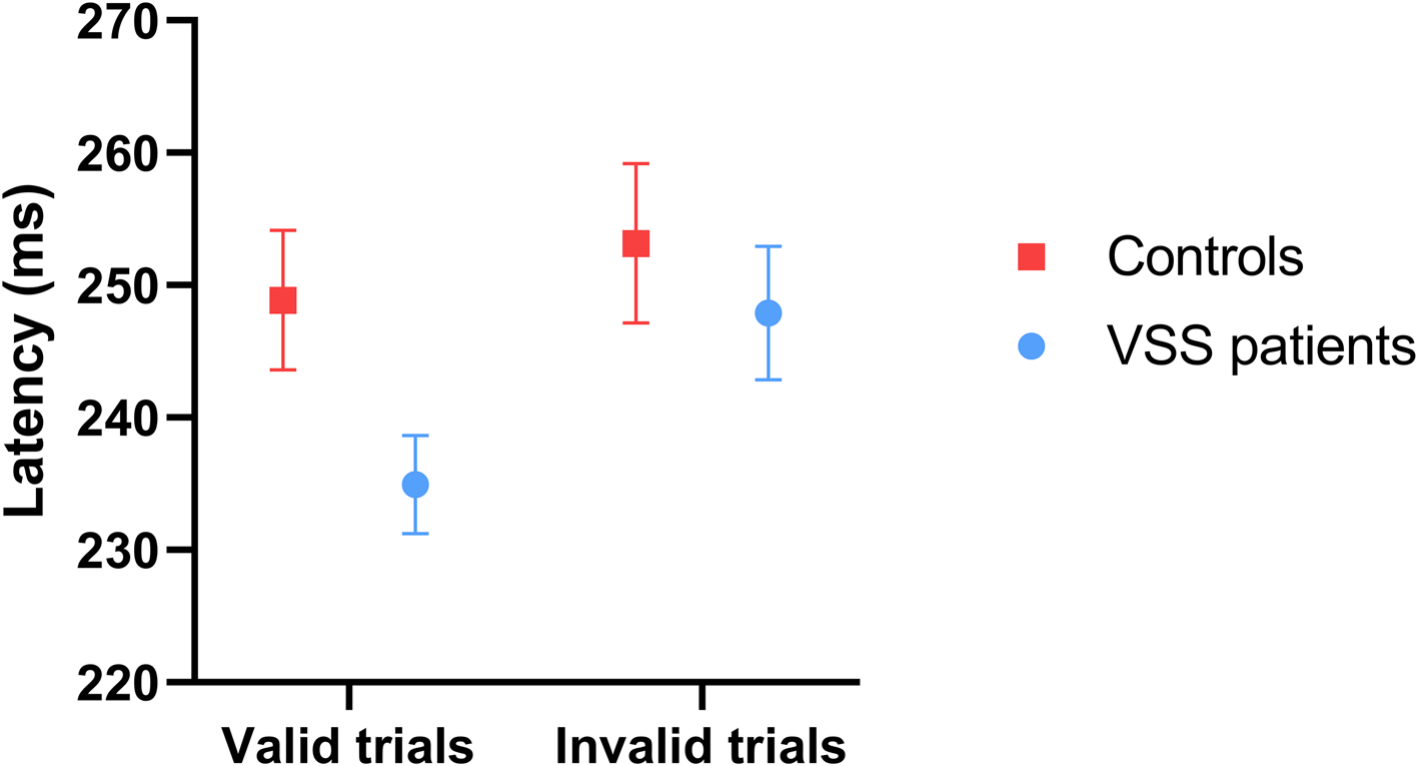
Control and VSS patient latencies on the endogenously cued saccade task. *Note.* Mean latencies of controls and VSS patients are shown for valid and invalid trials on the endogenously cued saccade task. Error bars represent standard errors. VSS: Visual snow syndrome**.**

A significant main effect of trial type was found, (*F*(1,94)=16.88, *p*<0.001, ηp=0.152, CI [235.66, 238.14]) with latencies for valid trials significantly shorter than latencies for invalid trials (mean difference = 9.7065). A significant trial type × group interaction was found, (*F*(1,94)=4.25, *p*=0.042, ηp=0.043), with post hoc analyses revealing that while both groups demonstrated the same relationship between valid and invalid trials (invalid > valid: controls: (*t*(35)=− 2.03, *p*=0.05, *d*=0.13, 95% CI [− 8.6, 0.0]); VSS: (*t*(59)=− 4.33, *p*<0.00, *d*=0.38, 95% CI [− 18.93, − 6.97])), valid trial latencies were significantly shorter for VSS patients than controls (*F*(1,94)=4.91, *p*=0.029, *d*=.46, 95% CI [233.99, 246.32]). This was reflected in the significantly larger cue effect found for VSS patients compared to controls, *t*(93.41)=− 2.36, *p*=0.02, *d*=.43, CI [− 15.93, − 1.38], see Table 4. In contrast, invalid trial latencies were not significantly different between groups. Mean latencies of controls and patients for valid and invalid trials can be viewed in Figure 3.

For valid trial latencies, ROC curve analysis indicated that the area under the curve was 0.63 (SE=0.06, *p*=0.036, CI [0.516, 0.741]).

#### Error rate

**Figure 4:**
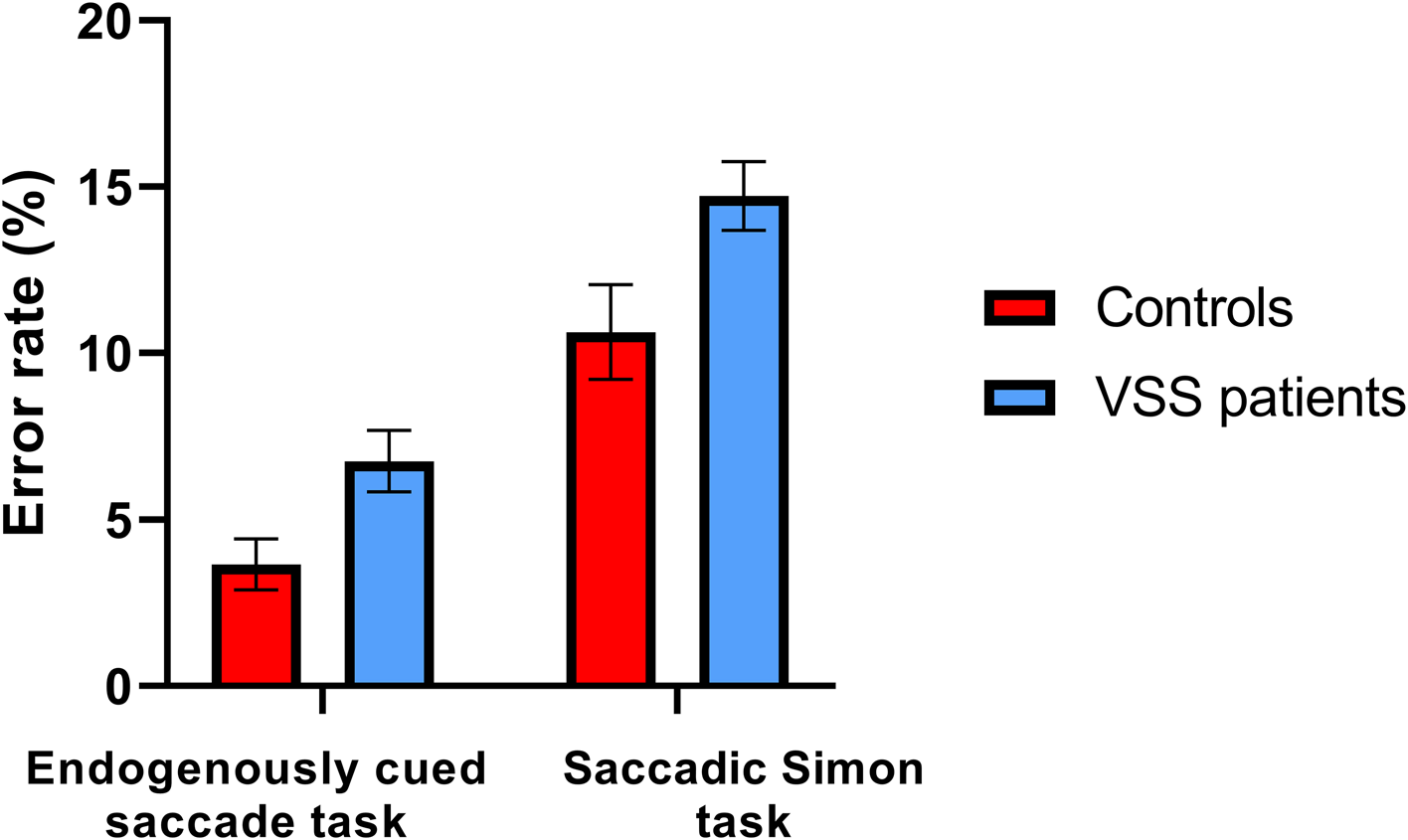
Control and VSS error rates on the endogenously cued saccade task and saccadic Simon task. *Note.* Mean error rates of controls and VSS patients are shown for the endogenously cued saccade task and saccadic Simon task. Error bars represent standard errors. VSS: Visual snow syndrome**.**

VSS patients generated a significantly higher rate of errors than controls (*F*(1,94)=5.54, *p*=0.021, *d*=0.52, 95% CI [4.3, 6.9]), see Table 4. Mean error rates for controls and patients are displayed in Figure 4.

ROC curve analysis indicated that the area under the curve for errors was 0.66 (SE=0.07, *p*=0.02, CI [0.53, 0.79]).

### Saccadic Simon task

#### Latency

A significant main effect of current trial was found, with latencies for congruent trials significantly shorter than latencies for incongruent trials (*F*(1,92)=5.55, *p*=0.021, ηp=0.57, 95% CI [479.29, 512.43]). On average, incongruent trial latencies were 12.13ms slower than congruent trials. This effect was consistent across groups, with no group × current trial interaction.

A significant previous trial × current trial interaction was found (*F*(1,92)=5.17, *p*=0.025, ηp=0.53, CI [466.41, 521.94]); this is in line with the expectation that only trials preceded by a congruent trial should show latency modulation. Specifically, the Simon effect, or longer latencies for incongruent versus congruent trials, was only seen for trials following a congruent trial. This was consistent across groups with no group × previous trial × current trial interaction found. No other interaction or main effect of group was found.

#### Error rate

A significant main effect of current trial was found, with more errors occurring on incongruent than congruent trials *(F*(1,92)=5.43, *p*=0.022, ηp=0.56, CI [11.53, 16.24]); this was found to be consistent across groups with no current trial × group interaction found. A significant main effect of previous trial was also found (*F*(1,92)=9.89, *p*=0.002, ηp=0.97, CI [10, 13.72]), with less errors occurring on trials following an incongruent trial than a congruent trial; again, this was found to be consistent across groups with no previous trial × group interaction found.

A significant previous trial × current trial interaction was present (*F*(1,92)=34.58, *p*<0.001, ηp=0.273, CI [8.75, 12.37], with more errors occurring when the current trial type was different to the previous trial type (i.e. a congruent trial following an incongruent, or an incongruent trial following a congruent trial). This pattern was consistent across groups with no previous trial × current trial × group interaction found.

A significant main effect of group was found, with VSS patients exhibiting a higher error rate, irrespective of current or previous trial type, compared to controls, (*F*(1,92)=4.43, *p*=0.038, ηp=0.046, 95% CI [12.75, 16.75]).

ROC curve analysis indicated that the area under the curve for errors was 0.66 (SE=0.07, *p*=0.019, CI [.529, .788]).

## Discussion

VSS is a complex disorder of sensory processing, manifesting in a range of visual and non-visual symptoms. Here we sought to determine whether the more rapid stimulus-driven shift of attention we identified previously is evident when attention is internally or endogenously redirected. For the endogenously cued saccade task we revealed a normal *cue effect* in VSS (i.e. shorter latencies for validly cued saccades). However, the magnitude of this effect was significantly larger for VSS patients, a consequence of shorter validly cued saccade latencies. VSS patients also generated significantly more erroneous saccades corresponding with the cue rather than the target. For the saccadic Simon task we revealed a normal *Simon effect* for both groups (i.e. the latency of an incongruent trial was longer than a congruent trial for those trials following a correctly executed congruent trial). Again, VSS patients generated significantly more erroneous saccades, although these errors bore no relationship to the cue stimulus.

These results demonstrate that endogenously driven shifts of attention more strongly influence saccade-related activity in VSS patients compared to controls, leading to greater difficulty controlling a relatively rapid shift of attention. A significant body of research has shown that saccades and attention share an obligatory relationship, both functionally and neuroanatomically^25^. Directing attention towards a specific spatial location alters the activity of populations of neurons that process stimuli at that location, as well as neurons that generate a saccade towards that location, influencing the direction and timing of a saccade^26^. This occurs whether attention is captured by a suddenly appearing visual event (exogenously driven) or redirected internally (endogenously driven).

For the endogenously cued saccade task, the preponderance of a centrally presented arrow that correctly predicts the location of an upcoming target, results in a pre-emptive shift of attention towards the probable location of an upcoming target. Consequently, visual processing is prioritised at the cued location and saccade-related activity enhanced, resulting in relatively shorter latencies^27^. Conversely, latencies for invalidly cued saccades are relatively prolonged due to the added requirements of reorienting attention away from the cued location and generating a saccade in the opposite direction^17,28^. Correct performance of this task also requires the suppression or inhibition of saccade-related activity evoked by a shift of attention until a visual target appears^17^.

Shorter latency saccades to validly cued targets suggest that for VSS patients, the balance between saccade-related and inhibition-related activity is shifted compared to controls such that, saccade-related activity sits relatively closer to threshold for release once a corresponding target appears. While an increased proportion of errors suggests that this saccade-related activity breaches threshold for the release more readily^17^, it is not possible from this data to determine whether this imbalance is due to reduced inhibitory or increased saccade-related activity. However, we have previously shown that a speeded response is unlikely to be due to executive failure^12^, and therefore presume that this reflects a stronger response to the cue, corresponding with greater saccade-related activity at the cued location.

For VSS patients, when saccade-related activity provoked by the cue is successfully inhibited and an error prevented, a corresponding saccade to a target is generated more quickly—significantly so for validly cued trials. Although not significantly different to controls, invalidly cued saccade latencies were also shorter, potentially reflecting the added difficulty in redirecting saccade-related activity away from the cued location. Notably, the differences seen between VSS and control latencies for cued saccades, were considerably smaller than previously reported for un-cued saccades^12^, suggesting a delay caused by greater difficulty balancing saccade-related and inhibition-related activity, a consequence of the cue. This may reflect additional inhibition-related activity required to maintain this balance in VSS.

The results of our saccadic Simon task appear consistent with this notion. This task requires interpretation of an abstract symbol presented in one of two locations. Here a participant must maintain fixation at centre, and orient attention to both peripheral stimuli to determine, (1) whether the *task* stimulus is a circle or square, and (2) based on this information, decide whether to look left or right. Consequently, and as for the endogenously cued task, a shift of attention results in increased saccade-related activity for neurons encoding the location of that shift, irrespective of direction, and the need to inhibit any response until a decision is made regarding the correct saccade location. VSS patients generated more errors, which like the endogenously cued saccade task, may be perceived as a premature breach of threshold for release of a saccade following the re-orienting of attention. Unlike the endogenously cued saccade task, these errors were not related directly to the cue stimulus, but likely the indiscriminate exploration of task stimuli; again, this suggests that saccade-related activity more readily breaches threshold for release in VSS. Given that a normal Simon effect was found for VSS patients, it is possible that these errors are *not* due to poor frontally-mediated inhibitory control as seen with other patient populations, such as Parkinson’s disease^29^ and schizophrenia^30^, who exhibit significant differences between congruent and incongruent trial latencies. Instead we attribute these errors to increased saccade-related activity, a consequence of a shift of attention^22^. However, we do acknowledge that the increased number of errors for VSS patients may have reduced our capacity to determine any differential effect of trial type (i.e. a difference in the size of the Simon effect) and temper this proposal accordingly.

VSS latencies on the Simon task were not differentially impacted by cue presentation. This is similar to results from our previous study where antisaccade latencies^12^, which also require a volitional response following an informative cue, were comparable to controls. Again, although VSS saccade latencies were not significantly different to control latencies, they were relatively prolonged compared to controls. This too may reflect a subtle delay caused by the requirement of maintaining saccade-related activity at sub-threshold levels.

Overall, these results confirm our proposal that attentional changes underpin the visual processing changes we revealed previously, demonstrating that the differences identified in tasks that exogenously direct attention, are similarly found in those that endogenously direct attention. Specifically, both exogenous and endogenous shifts of attention appear to more strongly increase saccade-related activity in VSS, resulting in increased errors and altered saccade latencies. As attention networks are complex and widespread in the cortex, the precise nature and location of dysfunction in VSS cannot be determined from these saccadic tasks alone. However, inferences may be made, and the combination of latency and error differences are consistent with earlier, lower level changes in visual processing, and not later executive processing of visual stimuli.

This heightened activation by either a visual stimulus or endogenous shift of attention may reflect increased excitability directly within visual processing regions, as theorised to occur in VSS^4,9,11,31^. Lauschke, et al.^2^ proposed that cortical hyperexcitability may be associated with disruption to thalamocortical communication. Although simplistic, the thalamus acts as a gatekeeper for the flow of sensory information to cortex, and influences the ongoing cortical processing of sensory information through recurrent feedback loops^32,33^. We have previously shown that perceptual surround suppression is altered in those with VSS^11^. While the mechanisms involved in neuronal surround suppression in the primary visual cortex involve both feedforward, horizontal, and feedback connections, the thalamocortical feedforward connections can strongly influence suppression strength^34^. Hence, if thalamocortical communication is altered in VSS, alterations to perceptual surround suppression would be a predicted outcome. In further support of this theory, tinnitus and migraine, which frequently occur alongside VSS, have also been associated with abnormal thalamocortical communication^35^.

These factors alongside our own findings lead us to hypothesise that a disruption to thalamocortical networks, alongside heightened excitability of associated cortical regions, may underlie the emergence of VSS by impacting the filtering and processing of sensory information. The ocular motor signature of VSS identified by this and our earlier studies may not only provide a quantifiable measure of dysfunction in VSS that can be used to monitor the efficacy of any future treatments, but may also be used to inform future investigation of the mechanisms disrupted in VSS, guided by our hypothesis of disruption to thalamocortical networks.
